# Development and Evaluation of a Live Attenuated Egg-Based Camelpox Vaccine

**DOI:** 10.3389/fvets.2021.721023

**Published:** 2021-08-16

**Authors:** Kuandyk Zhugunissov, Sanat Kilibayev, Muratbay Mambetaliyev, Kunsulu Zakarya, Markhabat Kassenov, Yergaliy Abduraimov, Yerbol Bulatov, Moldir Azanbekova, Zharkinay Absatova, Khairulla Abeuov, Ryspek Nurgaziev, Gourapura J. Renukaradhya, Kairat Tabynov

**Affiliations:** ^1^Laboratory Collection of Microorganisms, Research Institute for Biological Safety Problems, Gvardeiskiy, Kazakhstan; ^2^Testing Laboratory Control of Technology and Biological Products, Research Institute for Biological Safety Problems, Gvardeiskiy, Kazakhstan; ^3^Laboratory Cultivation of Microorganisms, Research Institute for Biological Safety Problems, Gvardeiskiy, Kazakhstan; ^4^Laboratory for Diagnostics of Infectious Diseases, Research Institute for Biological Safety Problems, Gvardeiskiy, Kazakhstan; ^5^Faculty of Veterinary Medicine and Biotechnology, Kyrgyz National Agrarian University Named After K.I. Skryabin, Bishkek, Kyrgyzstan; ^6^Department of Animal Sciences, Center for Food Animal Health, College of Food Agricultural and Environmental Sciences, The Ohio State University, Wooster, OH, United States; ^7^International Center for Vaccinology, Kazakh National Agrarian University, Almaty, Kazakhstan; ^8^Preclinical Research Laboratory With Vivarium, M. Aikimbayev National Scientific Center for Especially Dangerous Infections, Almaty, Kazakhstan

**Keywords:** camelpox virus, vaccine, attenuation, safety, immunogenicity, protective efficacy, camel

## Abstract

Camelpox is an infectious viral disease of camels reported in all the camel-breeding areas of Africa, north of the equator, the Middle East and Asia. It causes huge economic loss to the camel industry. We developed a live camelpox virus vaccine candidate using an attenuated strain and evaluated its safety, immunogenicity and protective efficacy in camels. The attenuated virus strain was generated from the camelpox wild-type strain M-96 by 40 consecutive passages on the chorioallantoic membrane of 11-day-old embryonated chicken eggs, henceforth called KM-40 strain. Reversion to virulence of the KM-40 strain was evaluated in camels by three serial passages, confirmed its inability to revert to virulence and its overdose administration was also found safe. Studies of immunogenicity and protective efficacy of the candidate vaccine KM-40 strain in camels was carried out using the dose of 5 x 10^4.0^ EID_50_. Our data showed complete protection against the challenge infection using the virulent wild-type camelpox virus strain M-96 (dose of 10^5.0^ EID_50_) which was evaluated at 1, 3, 6 and 12 months post vaccination. In summary, our candidate live attenuated egg-based camelpox vaccine strain KM-40 was found safe, protective, and thus has the potential to use safely in field conditions.

## Introduction

Camelpox is a widespread infectious viral disease of camels. It occurs throughout the camel-breeding areas of Africa, north of the equator, the Middle East and Asia, causing economic impact through loss of production and death (1). Its etiological agent is Camelpox virus (CMLV), belongs to the genus Orthopoxvirus (OPV) of the family Poxviridae (2). Phylogenetic analysis shows that among OPVs, CMLV is the closest strain to variola virus (VARV), the causative agent of smallpox (3), although each virus exhibits a strictly narrow host range.

Camelpox is characterized by fever, local or generalized pox lesions on the skin and in the mucous membranes of the mouth, respiratory and digestive tracts. The clinical manifestations range from inapparent infection to mild, moderate, and less commonly, severe systemic infection and sometimes death. Severe camelpox outbreaks in naïve young camelids cause high mortalities (1, 4). Various studies have demonstrated that the incidence of camelpox outbreaks increased during rainy seasons (5) with the appearance of more severe form of the disease, while milder form occurs during the dry season (6). The incidence and case fatality rate (CFR) are mostly higher in male camels than females. The mortality in adult animals is ranged from 10 to 28 % and in young animals 25–100 % (7). The variation in the severity of clinical signs possibly reflects differences between the strains of CMLV (5). The infection has been described as a possible zoonosis with three human cases identified and confirmed in India (8).

The disease is endemic in the Middle East (Iran, Iraq, Saudi Arabia, United Arab Emirates (UAE) and Yemen), in Asia (India, Afghanistan, and Pakistan), in Africa (Algeria, Egypt, Kenya, Mauretania, Niger, Somalia and Morocco, Ethiopia, Oman, Sudan) and in the southern parts of former the Union of Soviet Socialist Republics (USSR) (9–12). The epizootics of this disease in Kazakhstan were observed in 1930 and 1942–1943 (13). Since 1965, camelpox has reappeared in Kazakhstan in the farms of Guryevsk region in the autumn, winter and spring seasons, and the recent outbreaks were registered in 1996 (14) and 2020 (15) in Mangistau region. To mitigate the outbreaks of camelpox (1996) in the Mangistau region of the Republic of Kazakhstan, a Russian live vaccine based on a strain of vaccinia virus was used (data not published).

An ideal tool to control camelpox is through vaccination. Currently, worldwide there are four camelpox vaccines (10, 16), of which two have been evaluated and commercialized. They contain the following CMLV strains: Jouf-78 (17), VD47/25 (18), Ducapox 298/ 89 (19) and CMLV-T8 (20). However, non-availability of a potent and cost-effective camelpox vaccine in the Republic of Kazakhstan and in many camel-rearing developing and underdeveloped countries has been the major constraint in control of the disease outbreaks. Thus, we developed a live attenuated egg-based camelpox vaccine using an available local field strain and evaluated its safety and efficacy in camels.

## Materials and Methods

### Camelpox Virus Strains

We used an attenuated strain KM-40 (21) of CMLV obtained after 40 consecutive passages on 11-day-old embryonated chicken eggs (ECE) of the local virulent field strain M-96 (21, 22). The growth kinetics of the attenuated virus is same as the wildtype parent strain (21). The virulent wild-type strain M-96 (GenBank # AF438165.1) isolated from sick camels during a field outbreak that registered in the Mangistau region of Kazakhstan in 1996 (14) was used in our challenge trial.

### Vaccine Preparation

The KM-40 strain containing suspension with an activity of > 10^6.0^ EID_50_/ml on CAM of ECE (Supplementary Materials) was prepared to use as a vaccine candidate. A mixture of 1:1 ratio of attenuated virus suspension with sterile stabilizing medium containing 13% peptone from casein (Sigma-Aldrich, Germany) was aliquoted into 1 ml ampoules (20 doses), lyophilized, and stored at 4°C. Master seed, working seed and the experimental vaccine batches were prepared using ECE according to the World Organization for Animal Health (OIE) principles for the production of veterinary vaccines (1). Methods of infection of ECE, titration of virus infectivity, preparation of stabilizing medium, sublimation drying, and preparation of 50% glycerol solution are presented in the Supplementary Materials.

### Animals and Bioethics

Forty-five male camels of Camelus bactrianus (*n* = 23) and Camelus dromedaries (*n* = 22) breeds aged 8–12 months obtained from farms in the southern region of Kazakhstan free from any infectious diseases and seronegative for CMLV were used in the study. Animals were kept under quarantine for 4 weeks before used in the experiment by monitoring the body temperature, clinical examination and testing of serum samples for the presence of CMLV specific viral neutralizing antibodies by serum neutralization test (SNT). Animals seronegative to CMLV were used in the experiment. During the immunization phase the animals were kept in animal house of the Research Institute for Biological Safety Problems (RIBSP), and later transferred to the ABSL-2 laboratory for virus challenge studies. Access to feed and water for the animals was provided *ad libitum* in both the facilities. The camels were examined by a certified veterinarian for dermatotropic diseases such as foot and mouth disease, pox-like disease (auzdyk), trichophyton verrucosum, etc. All camels were treated against ticks and internal parasites using Ivermectin.

This study was performed in compliance with national and international laws and guidelines on animal handling [the U.K. Animals Scientific Procedures Act 1986 and guidelines of EU Directive 2010/63/EU for animal experiments). The experimental protocol was approved by the Committee on the Ethics of Animal Experiments of the RIBSP of the Science Committee of the Ministry of Education and Science of the Republic of Kazakhstan (permit number: 0414/002 and 0121/013).

### Vaccine Safety Evaluation

**Figure 1:**
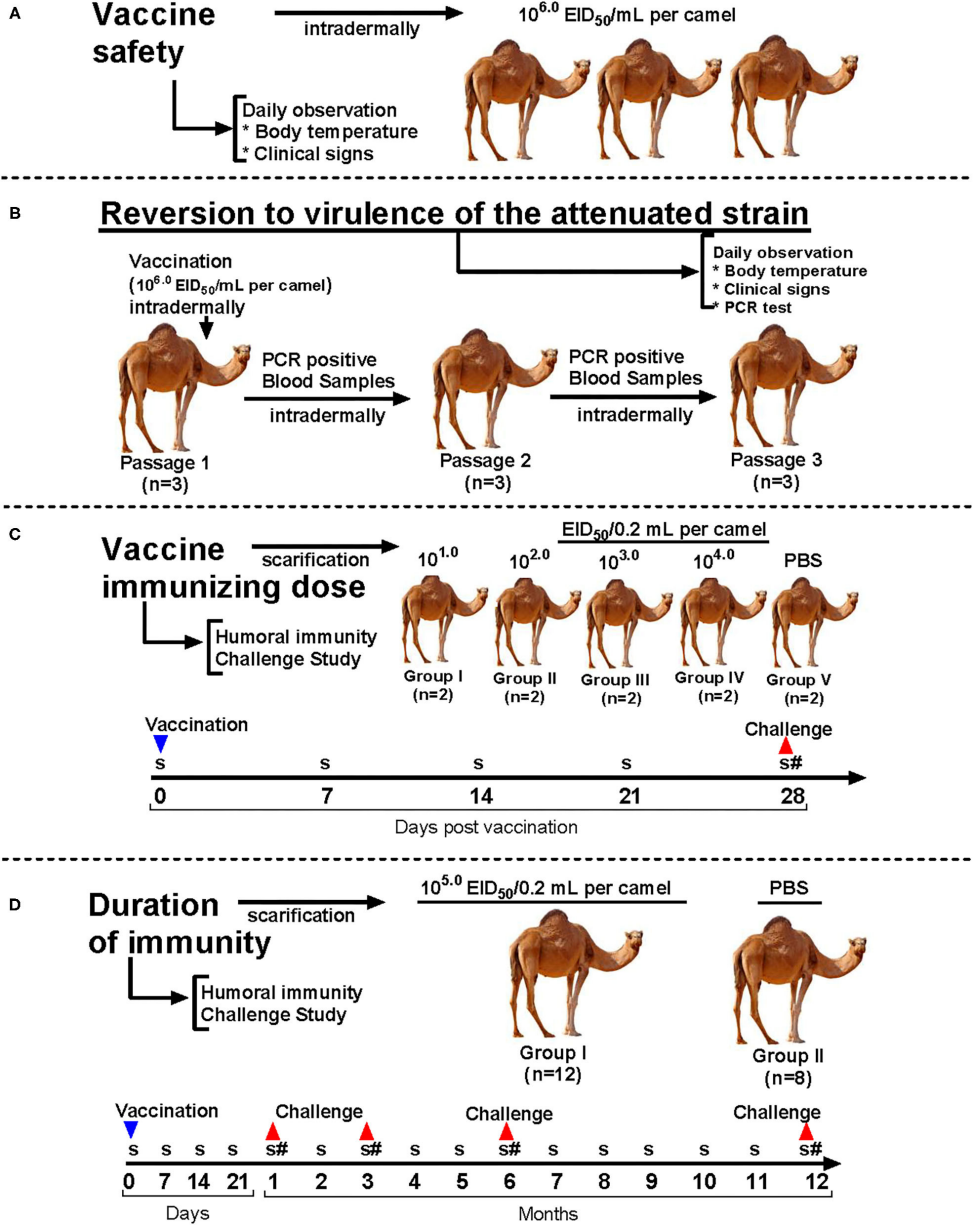
Experimental design of the study. **(A)** Vaccine safety evaluation; **(B)** Reversion to virulence evaluation; **(C)** Vaccine immunizing dose and protectiveness evaluation; **(D)** Duration of the immune response and protective efficacy evaluation. **S**, serum collection; **#**, challenge.

Vaccine safety was tested by injection of the candidate vaccine at a dose of 10^6.0^ EID_50_ in 1 mL (20 × higher than the actual vaccine dose) intradermally to three camels (Figure 1A). The control animals (*n* = 3) were administered with 1 mL of phosphate-buffered saline (PBS) intradermally. After vaccination, body temperature was monitored daily and monitored for 14 days. Animals showing clinical signs (increased body temperature, the appearance of vesicles and papules, or any condition that prevented food or water intake) were euthanized. Euthanasia of camels was carried out according to AVMA Guidelines for the Euthanasia of Animals: 2020 Edition (23).

### Reversion to Virulence Evaluation

Analysis of reversion to virulence of attenuated strain KM-40 of CMLV was conducted according to OIE recommendations for live vaccines by serial passaging on susceptible animals (24). We used nine seronegative camels for CMLV, divided into three groups (*n* = 3 per group). Each group was kept separately in ABSL-2 rooms to avoid any contact among the groups. The first group was administered with the vaccine dose of 10^6.0^ EID_50_ diluted in PBS intradermally. For determination of viremia, 2–14 days after virus inoculation the blood samples were collected from febrile animals in lithium heparin and EDTA tubes (Becton, Dickinson and Co., USA). The blood samples were tested for the presence of viral DNA by using a commercial RT-PCR kit (Genesig^®^ Advanced PCR kit, Primerdesign™ Ltd, UK), as well as for the presence of live CMLV by infection on the CAM of ECE. One mL inoculum was administered to the second group of seronegative camels. Similar testing was carried out after the third viral passage (Figure 1B). Animals were observed to evaluate their health status and viremia for 14 days.

### Evaluation of Immunizing Dose and Protective Efficacy of the Vaccine

Ten experimental camels were divided into five groups (I, II, III, IV, and V) of two animals each. Each animal in Groups I–IV received the vaccine by scarification of the skin and application of the vaccine diluted in sterile 50% glycerol with a dose of 10^1.0^, 10^2.0^, 10^3.0^, and 10^4.0^ EID_50_/0.2 ml, respectively. Group V was administered with PBS containing 50% glycerol served as a control (Table 1). Vaccinated animals were monitored daily and at 14, 21, and 28 days after vaccination the blood samples were collected from all animals to study virus neutralization titers. Animals were challenged with a virulent wild-type virus at a dose of 10^5.0^ EID_50_/0.2 mL by scarification of the skin and application in a shaved area on the hind limbs at 28 days after vaccination to study the protective efficacy (Figure 1C).

**Table 1:** Vaccine immunizing dose and protective efficacy analysis.

**Group (n=2)**	**Vaccine dose**	**Challenge dose**
	**(EID_**50**_/0.2 mL)**	**(EID_**50**_/0.2 mL)**
I	1 x 10^1.0^	1 x 10^5.0^
II	1 x 10^2.0^	1 x 10^5.0^
III	1 x 10^3.0^	1 x 10^5.0^
IV	1 x 10^4.0^	1 x 10^5.0^
V	PBS in 50% glycerol	1 x 10^5.0^

*PBS, phosphate-buffered saline*.

*EID, embryo infectious dose*.

### Evaluation of Duration of the Immune Response and Protective Efficacy

Twenty clinically healthy 8–12 months old camels seronegative for CMLV were used. Animals were divided into two groups: vaccinated (Group 1; *n* = 12) and control (Group 2; *n* = 8). Animals in Group 1 were vaccinated with lyophilized vaccine resuspended with 50% glycerol and using a field dose of 5 x 10^4.0^ EID_50_ in a final volume of 0.2 mL by scarification of the hairless area of the hind limbs. Control animals (Group 2) were similarly administered with PBS (Table 2). Vaccinated animals were monitored daily and at 7, 14, 21, 30 days and subsequently every month for 12 months post vaccination the blood samples were collected and analyzed for virus neutralization titers. Furthermore, camels were challenged at 1, 3, 6 and 12 months post vaccination to study the protective efficacy (Figure 1D). This was performed by scarification of the skin and application of the M-96 strain of CMLV at a dose of 10^5.0^ EID_50_/mL in a shaved area on the hind limbs (25). Lyophilized wild-type strain M-96 obtained from the Repository of agents of particularly dangerous pathogens of the RIBSP was reconstituted with 50% glycerol and applied by the method indicated above. The animals were monitored for 14 days and observed for general health, body temperature and clinical signs of camelpox.

**Table 2:** Evaluation of duration of immune response and protective efficacy.

**Group**	**Vaccine dose**	**Challenge dose**
	**(EID_**50**_/0.2 mL)**	**(EID_**50**_/0.2 mL)**
I (*n* = 12)	5 x 10^4.0^	1 x 10^5.0^
II (*n* = 8)	PBS in 50% glycerol	1 x 10^5.0^

*PBS, phosphate-buffered saline*.

*EID, embryo infectious dose*.

### DNA Extraction and Polymerase Chain Reaction

DNA extraction was performed by using QIAamp DNA Mini and blood Mini kits (Qiagen, Venlo, Netherlands) following manufacturer's instructions and samples were subjected to quantitative real-time PCR (qPCR).

Polymerase chain reaction (PCR) for detection of camelpox virus DNA in whole blood samples from infected camels was performed with a PCR kit according to the manufacturer's instructions (Genesig^®^ Advanced PCR kit, Primerdesign™ Ltd, UK). The following PCR cycling conditions were used on MJ dyad 96-well thermocyclers (Bio-Rad Inc. Hercules, CA): 95°C for 2 min, followed by 50 cycles of 95°C for 10 s and 60°C for 360 s.

### Histopathology

Paraffin sections from the tissue specimens were prepared and stained with Hematoxylin and Eosin as described previously (26). Briefly, the slides were deparaffinized in two changes of xylene, 15 min each and hydrate to water by descending grades of alcohol (95, 80, and 70%) 10 min each. Hematoxylin was added on slides for 15 min, washed in running tap water for 20 min and counterstained with Eosin for 2 min. The slides were dehydrated in 95% and absolute alcohol, two changes of 2 min each until excess Eosin was removed, and finally cleared using xylene (two changes of 2 min each).

### Virus Neutralization Test

Sera collected from experimental camels were heat-inactivated at 56°C for 30 min and tested for circulating neutralizing antibodies against CMLV using the standard serum neutralization test (SNT; constant-virus, diluted-serum) as described (1). The test sera were titrated by SNT against a fixed amount of camelpox virus (100 TCID_50_ [50% tissue culture infectious dose]).

### Statistical Analysis

The variance in protective efficacy of camel groups was compared by one-sided Fisher exact test. P < 0.05 was considered significant. Mean values of data are reported with standard errors of mean (SEM). Statistical analysis of all experimental data was performed using Graph Pad Prism Software Version 8.0 (Graph Pad Software Inc., La Jolla, CA, USA).

## Results

### Generation of a Live Attenuated Egg-Based Camelpox Vaccine

A stock virus was prepared from the wild-type M-96 strain of CMLV. The virus was identified by virus neutralization test and PCR. The virus was then serially propagated on the CAM of 11-day-old ECE. At passage 40 the EID_50_/ml of the virus was found approximately 10^6.0^/ml and the virus replication was characterized by the formation of pock lesions on CAM of ECE, which were small and opaque to white of various shapes and sizes (**Figure 3D**).

### Safety Evaluation of the Vaccine

**Figure 2:**
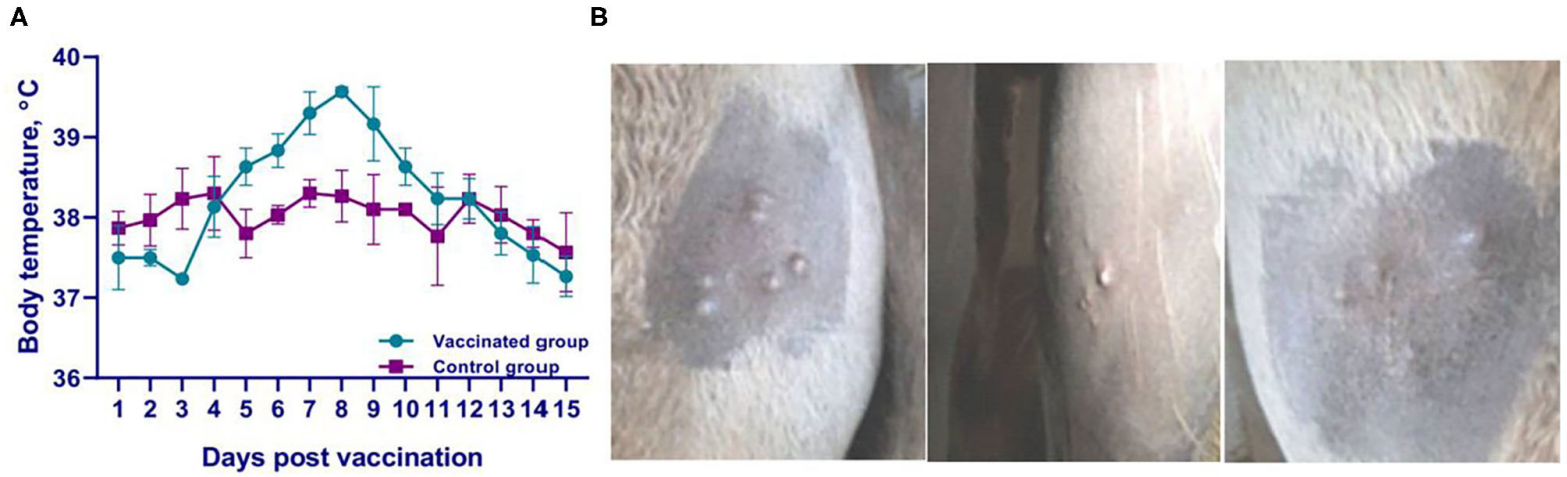
Clinical findings in camels during vaccine safety evaluation. **(A)** Temperature response of vaccinated and control groups of camels; **(B)** Response of camels to vaccine application. Skin swellings, 0.3-0.5 cm in size, disappeared by days 8–9 after virus administration.

All vaccinated (20 × concentrated vaccine dose; overdose study) animals did not show any clinical signs of camelpox for the entire observation period (14 days). Changes in clinical condition on days 6–7 were characterized only by a slight increase in body temperature among the vaccinates (Figure 2A), and the appearance of small skin swelling sized 0.3–0.5 cm at the vaccine application site (Figure 2B) disappeared by days 8–9 (Figure 2B). Daily measurements of body temperature, pulse rate and respiration rate of animals remained normal within the physiological norms (data not shown).

### Reversion to Virulence of the Attenuated Strain

**Figure 3:**
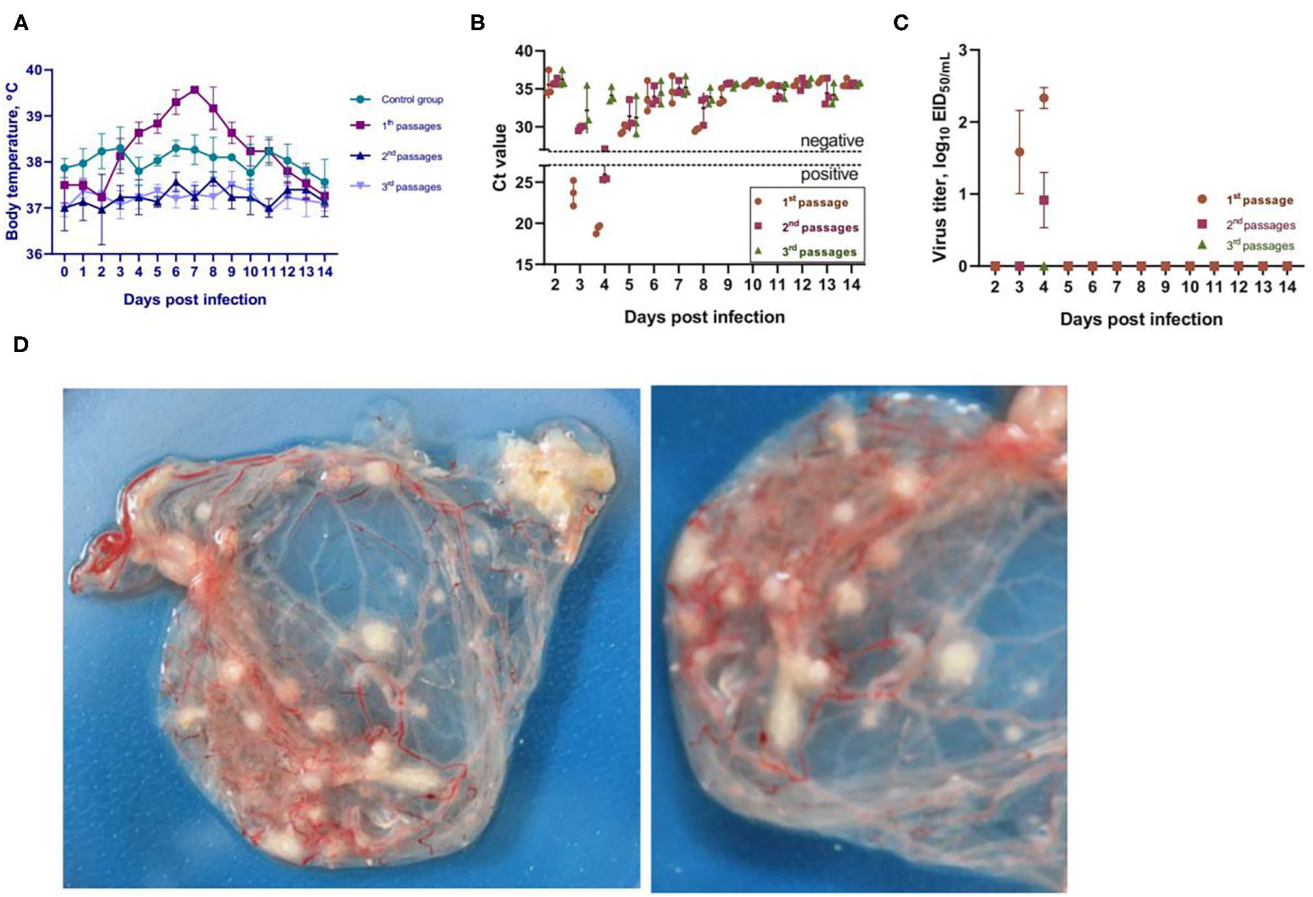
Reversibility testing of an attenuated KM-40 strain of camelpox virus. **(A)** Temperature response of vaccinated and control camels analyzed for vaccine strain reversibility. **(B)** PCR results for detection of viral DNA in the blood of infected animals. In the blood of infected camels sampled on days 3 and 4 after infection, viral DNA was detected at the first and second passages, whereas no virus DNA was detected in the blood of infected camels at the third passage. Ct values between 16 and 25 are positive, while Ct values ≥30 are negative. **(C)** Virus titer on CAM of the ECE in blood samples collected on days 3 and 4 after infection. **(D)** characteristic of the formation pock lesions on CAM of the ECE.

Reversion to virulence of the attenuated KM-40 vaccine strain of CMLV in camels at the first passage showed similar results (Figure 3A) as the vaccine safety test. The viremia was confirmed in blood samples by PCR at days 3 and 4 after infection (Figure 3B), and the virus was isolated from blood after first passage in ECE with a titer of 2.25 ± 0.16 log_10_ EID_50_/mL (Figures 3C,D). The viral DNA was detected in camels at the second passage level after infection only at day 4 (Figure 3B) with a viral titer of 0.7550 ± 0 ± 0.25 log_10_ EID_50_/mL (Figures 3C,D). While from the blood samples collected on days 3, 4, 5, 8, 9, and 10 after infection in 3rd virus passaged animals no viral DNA or virus could be isolated in the ECE. This was associated with the normal body temperature and absence of any clinical signs of camelpox in infected camels at the second and third passage level (Figure 3A). At the third passage, the clinical condition of the camels was like in animals of the second passage (Figure 3A). However, the virus neutralizing antibodies (VNA) were detected in sera of camels collected at 14, 21 and 28 days after first passage infection with titers ranging 0.75–4.5 log_2_, from second passage infection VNA titers ranging 0.5–3.0 log_2_, and from the third passage animals VNA were not detected in sera (data not shown). Overall, the attenuated vaccine strain KM-40 of the CMLV during all three serial passages in camels showed no pathogenic properties, and all the infected animals remained healthy during the observation period of 30 days (data not shown).

### Immunizing Dose and Protectiveness of the Vaccine

Immunizing dose and protectiveness of the vaccine were evaluated by vaccination of camels with different doses (10^1.0^, 10^2.0^, 10^3.0^, and 10^4.0^ EID_50_ in 0.2 mL) and weekly (14, 21, and 28 days), serum sampling followed by analysis of dynamics of VNA titers and challenge infection at day 28 with the virulent wild-type strain M-96 of CMLV (Table 3). Our results found that all the test groups of vaccinated animals did not exhibit any clinical signs of camelpox during the 14-day clinical observation period, and the body temperature was within the normal physiological range (data not shown). The level of specific VNA titers after immunization was higher in group IV compared to group III animals, while in groups I, II, and V animals VNA was not detectable (Table 3).

**Table 3:** Dynamics of VNA formation in sera after immunization with the vaccine and protectiveness after challenge with the virulent virus.

**Group**	**Vaccine dose**	**Serum collection**	**VNA titer**	**Challenge**
**(*n* = 2)**	**(EID_**50**_/0.2 mL)**	**(days)**	**(log_**2**_)**	**(1 x 10^**5.0**^EID_**50**_/ 0.2 mL)**
I	1 x 10^1.0^	14	0.00	positive
		21	0.00	
		28	0.00	
II	1 x 10^2.0^	14	0.00	positive
		21	0.00	
		28	0.00	
III	1 x 10^3.0^	14	0.00	positive
		21	1.00	
		28	1.50	
IV	1 x 10^4.0^	14	3.00	negative
		21	4.00	
		28	4.50	
V	PBS	14	0.00	positive
		21	0.00	
		28	0.00	

**Figure 4:**
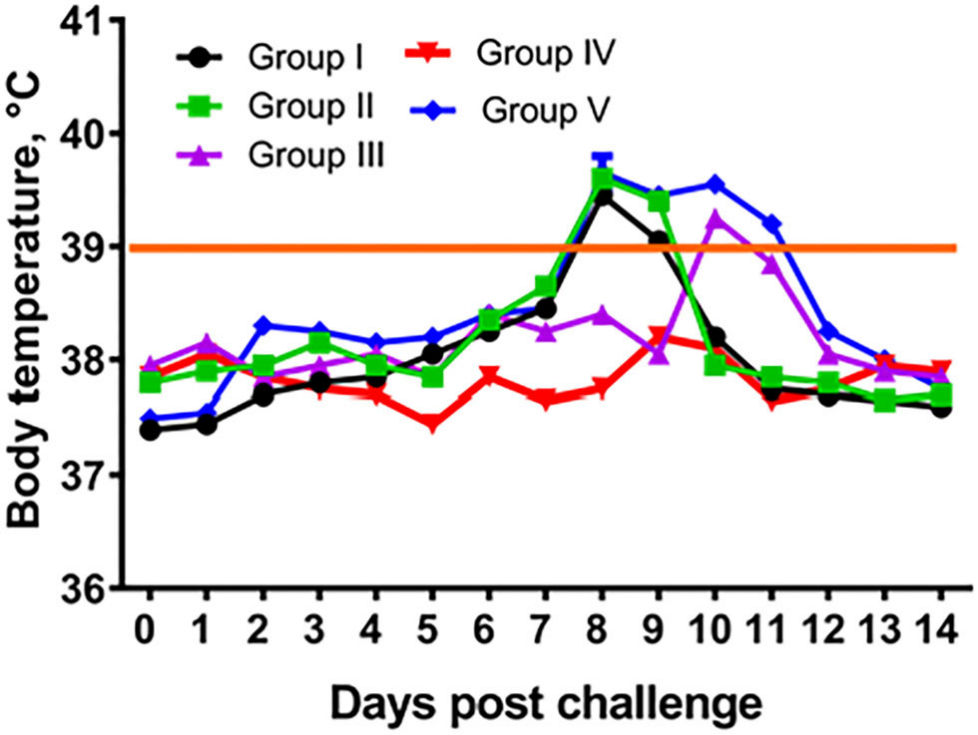
Temperature response of vaccinated camels after challenge with virulent wild-type virus. Group I – camels vaccinated using a dose 10^1.0^ EID_50_/0.2 mL; Group II – camels vaccinated using a dose 10^2.0^ EID_50_/0.2 mL; Group III – camels vaccinated using a dose 10^3.0^ EID_50_/0.2 mL; Group IV – camels vaccinated using a dose 10^4.0^ EID_50_/0.2 mL; Group I – control camels administered with PBS.

**Figure 5:**
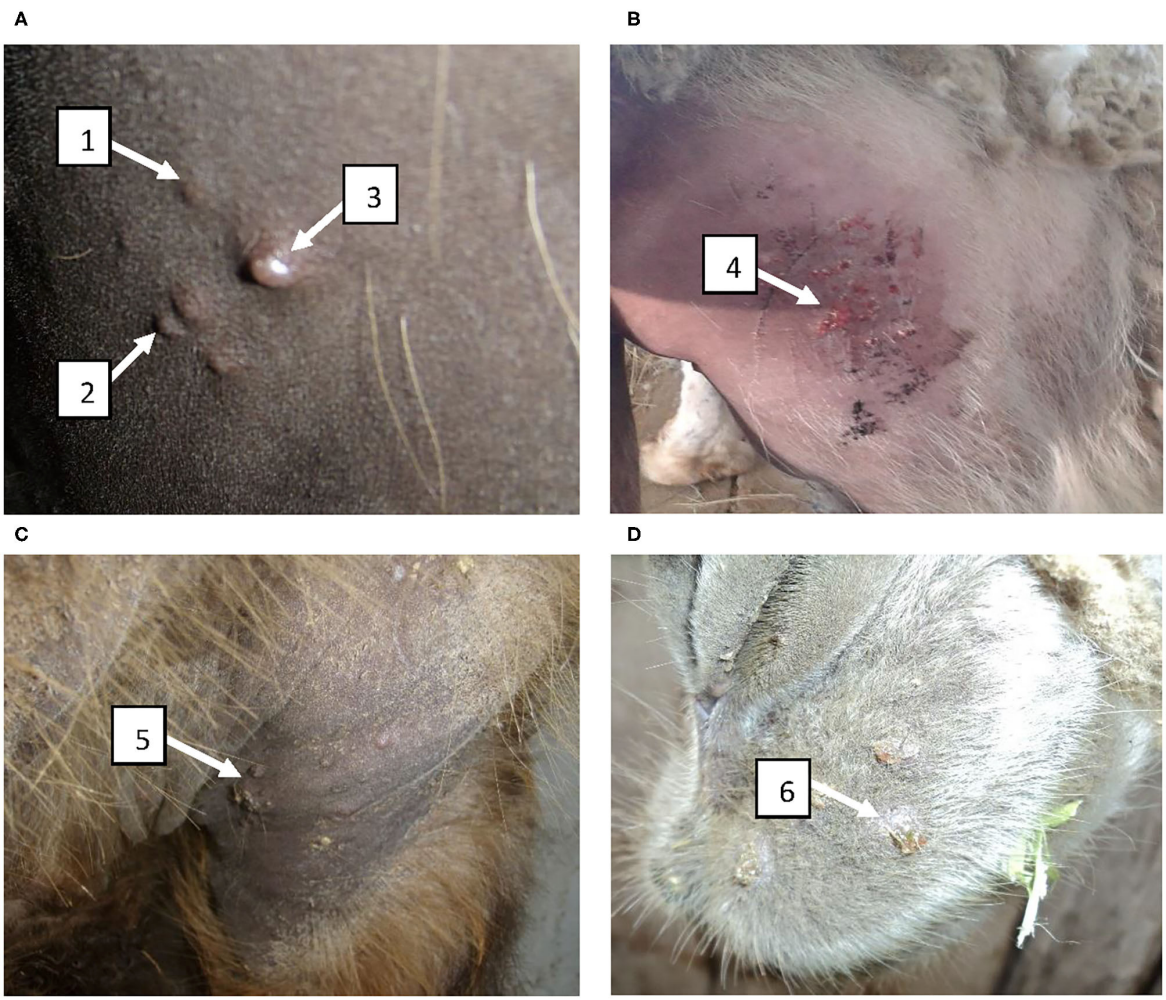
Clinical manifestation of disease in vaccinated camels infected with the virulent wild-type virus during determination of the immunizing dose of the vaccine. **(A)** Formation of papules, pustules and vesicles at the sites where the virus suspension was administered at 6–9 days after infection; **(B)** Formation of vesicles from pustules which became pox crusts by 11–14 days; **(C)** Development of pox lesions in the perineum at the anterior site at 11 days after infection; **(D)** Development of pox lesions on the lips at 11 days after infection. 1 – papule; 2 – pustule; 3 – vesicle; 4 – formation of pox crust; 5 – pox crust in the perineum of the front limbs of a camel; 6 – pox crust on the lips of a camel.

In groups I–III (immunized) and in group V (control, PBS) camels at post challenge infection days 8 to 11 we observed increased body temperature ranging 39.0–39.8°C. While group IV (10^4.0^ EID_50_ dose) camels' body temperature remained within the physiological level for the entire period of observation (Figure 4). In groups I–III and V at 6–9 days after infection, animals were depressed, papules, pustules and vesicles formed at the sites where virus suspension was administered (Figure 5A). Development of pox lesions on the lips (Figure 5D), in the perineum of the anterior (Figure 5D) and posterior limbs, in the groin area and throughout the head was noted only in groups I and V at 11 days after infection. On days 11–14, vesicles formed from the pustules turned into pox crusts (Figure 5B). By 12 days after infection, there was a decrease in body temperature to the physiological level (Figure 4). In contrast, group IV animals did not show any clinical signs of camelpox and the body temperature was within the physiological level during the 14-day observation period (Figure 4).

Our results suggested that the optimal dose of the candidate vaccine to immunize camels was 10^4.0^ EID_50_/0.2 mL (Group IV). But considering the data obtained on safety trials a dose equal to 5 x 10^4.0^ EID_50_ in 0.2 mL was selected, making a 5-fold reserve for various contingencies (climatic conditions, transportation, conditions of application, storage failure, etc.).

### Duration of the Immune Response and Protective Efficacy of the Vaccine

**Figure 6:**
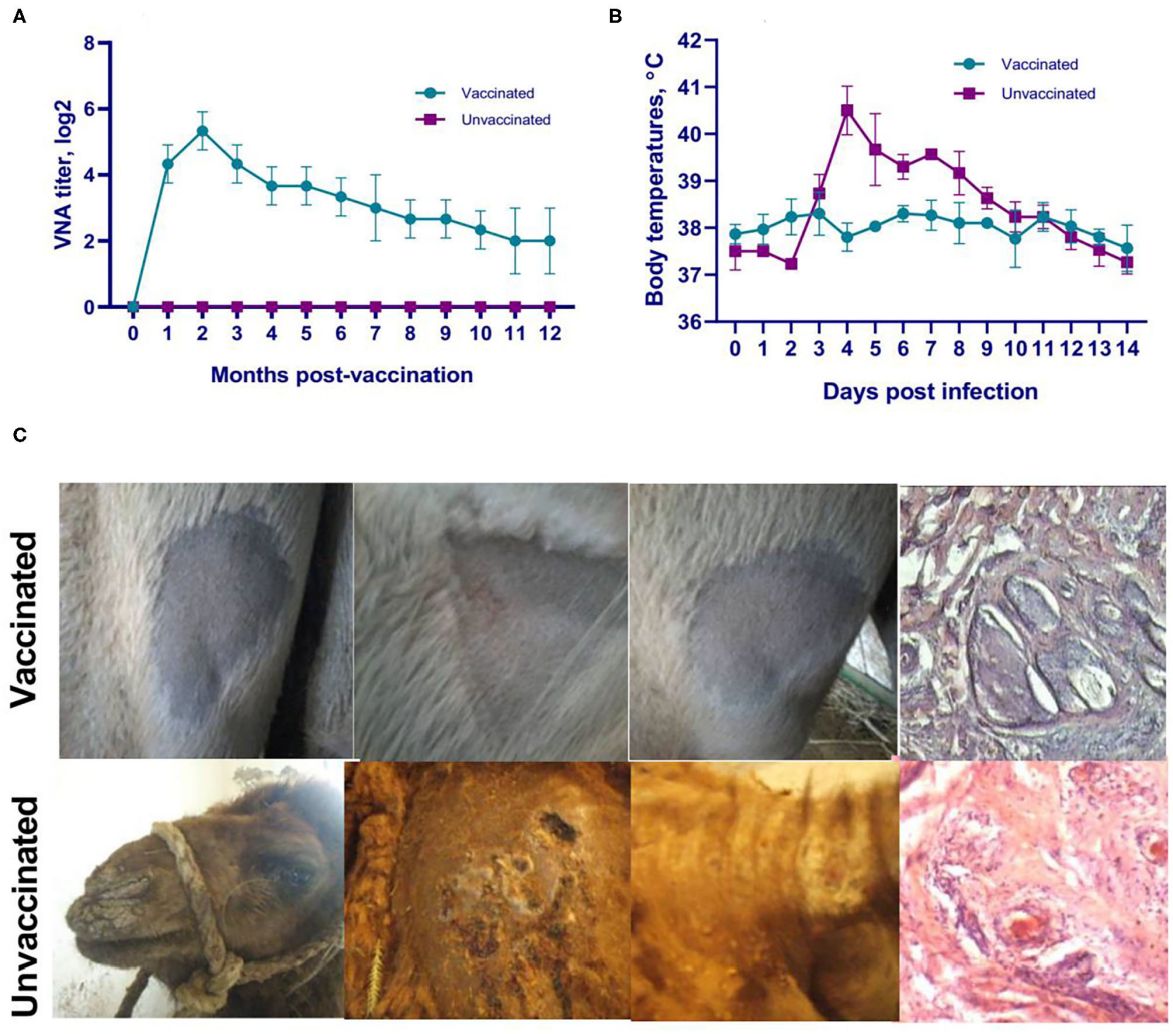
Evaluation of duration of the immune response and protective efficacy of the camelpox vaccine. **(A)** Dynamics of VNA formation in sera of vaccinated animals against camelpox. VNA are formed starting from days 6–7 and are detected even at 12 months in titers ensuring immunity of vaccinated animals to the virulent wild-type M-96 strain at the dose of 10^5.0^ EID_50_. **(B)** Temperature response of vaccinated and unvaccinated camels after challenge. **(C)** Cutaneous reaction and dermal histopathology of vaccinated and unvaccinated camels after challenge (multilayer squamous epithelium at the scarification area is relatively thin, the borders of the papillary and reticular layers of the skin are distinguishable, and primary and secondary follicles and other components of the skin base are visible). Formation of papules and vesicles in places of virus inoculation in unvaccinated animals was observed on 7–9 days, also on the skin of lips, outer part of nose wings, hind legs, head with subsequent formation of pox crusts on 10–14 days. The candidate vaccine induced an immune response that provided a statistically significant reduction in the severity of clinical signs of camelpox (*P* < 0.0001) and 100% protection (*P* < 0.0001).

Duration of the immune response and protective efficacy of the vaccine were evaluated by collection of blood samples monthly once and analysis of VNA titers and challenge infection trial. On day 7, the vaccinated group of camels (*n* = 12) showed VNA in serum in low titers ranging from 0.5880 ± 0 ± 0.08 log_2_ to 0.7550 ± 0 ± 0.00 log_2_ (data not shown). The dynamics of VNA formation at the 1st month after vaccination showed an increase in antibody titers averaging 4.3330 ± 0 ± 0.57 log_2_, which reached the peak titer of 5.3330 ± 0 ± 0.57 log_2_ at 2 months after vaccination. At the third month after vaccination, observed a decrease in antibody titers to 4.3330 ± 0 ± 0.57 log_2_, that gradually decreased to a titer of 2.0001 ± 1 ± 0.00 log_2_ at 12 months after immunization (Figure 6A). Comparison of mean antibody titers at months 1 and 3 with the maximum titer value at 2 months post vaccination interval was not statistically significant (*P* > 0.05), while the difference in VNA titers at the remaining months (4–12 months) with the above time point (2 months after vaccination) were significantly different (from *p* < 0.05 to *p* < 0.0001).

All vaccinated animals (*n* = 12) at all time points of infection (1, 3, 6, and 12 months after vaccination) remained healthy, without showing any clinical signs typical for camelpox. Moreover, at all points of viral challenge (1, 3, 6, and 12 months after vaccination), the vaccine showed 100% efficacy. In contrast, 100% unvaccinated camels (*n* = 8) showed an increase in body temperature by up to 40.8°C starting from 4 days after challenge (Figure 6B), and on 7–9 days after infection the manifestation of generalized clinical signs of the disease specific to camel pox such as oppression, refusal to take feed, formation of papules and vesicles in the sites of virus inoculation (Figure 6C), as well as on the skin of the lips (Figure 6C), the outer part of the wings of the nose, hind legs, and head were observed. At days 10–14, the affected areas were covered with pox crusts (Figure 6C).

### Histopathology of Vaccinated and Unvaccinated Camels After Challenge

The formation of pronounced forms of pox lesions was not observed at the site of skin scarification in vaccinated camels. At day 7 after challenge infection, multilayer squamous epithelium at the scarification area is relatively thin, the borders of the papillary and reticular layers of the skin are distinguishable, and primary and secondary follicles and other components of the skin base are visible (Figure 6C). In the skin and subcutaneous tissue, lesions in the form of pox or other manifestations of a focal inflammatory process are not observed. In unvaccinated camels, foci of inflammation around the deep part of the follicles consisting of eosinophils and mononuclear cells were observed on day 7 after challenge (Figure 6C). The affected skin epithelium was filled with inflammatory cells, and the lesion was also surrounded by a mixed inflammatory infiltrate that spread to the underlying muscular layer. At 21 days after infection, a large erosion appeared on the skin in which the squamous epithelium was lost and replaced by a crust (data not shown). In addition, no hair follicles remained in the underlying tissue and the adipose tissue was broken up by thin collagen threads containing few mononuclear inflammatory cells.

## Discussion

Camelpox was until recently considered a non-significant concern, but recently it has been seen as an emerging public health problem due to the increasing number of reported cases and outbreaks in camels (7). The occurrence of camelpox in humans, albeit quite rare, alerts the situation. There is a limited information on the production of vaccines against camelpox since its first inception about the concept of camelpox vaccine from former USSR (27). The knowledge of camelpox vaccine efficacy originates from field investigations using the commercialized CMLV-based vaccines. Of late to control camelpox, lacto-therapy (scarification of a mixture of milk and camelpox infected crusts) has been in use and practiced in Punjab (India), former USSR and Arabian Bedouin (7, 16). A vaccine based on vaccinia virus and variola detritus was used to prevent camelpox in the former USSR (27, 28). Supply of prophylactic and diagnostic products for camelpox in Kazakhstan were earlier procured mainly from bio-enterprises of Russia. After the collapse of the USSR, trade and economic ties with bio-enterprises of other republics ceased, but the threat of new outbreaks of camelpox continued.

Vaccination of camels with vaccinia virus (detritus) induces reliable immunity to natural infection. The vast majority of vaccinates (95%) show single pox lesions after 3–5 days at the site of application of the vaccinia virus. The process is benign and strictly localized, resulting in recovery by 12–20 days (27, 28). The use of vaccinia has been authorized by the Chief Veterinary Administration of the Ministry of Agriculture of the former USSR since 1969. Due to eradication of smallpox in 1980, public health officials stopped recommending the use of vaccinia virus to immunize animals due to potential risk to humans (10, 16). However, in 1996, during an outbreak in the Mangistau region of Kazakhstan it was forced to use vaccinia to eradicate camelpox (14). Camelpox vaccines based on the CMLV appeared after the elimination of smallpox. However, foreign commercial vaccines were not available in Kazakhstan. In this regard, we developed a domestic attenuated vaccine strain KM-40, which does not cause disease in camels. While all known attenuated strains of CMLV were obtained in cell cultures by serial passaging. For example, Jouf-78 vaccine strain passaged 80 times in camel kidney cell cultures and used in field testing (17); Ducapox 298/89 derived from O. Cameli strain passaged 96 times in Vero cells and used for camelpox-vaccine production (19); CP-NIG#114 derived from CP-NIG, Niger, and passaged 114 times in Vero cells (29), VD47/25 derived from VD47, Niger, and passaged 90 times in two cell types, used for camelpox vaccine production (18); CP-MAU#114 derived from CP-MAU, Mauritania, and passaged 114 times in cell culture Vero (30). Our KM-40 strain of CMLV is well adapted to grow on CAM of the ECE which cause pox plaques on the surface of the CAM on the third and fifth days, and the cultivation period is shortened by 2 days compared to the first passage (data not shown). Due to limited capacity, we did not perform genome sequencing of the attenuated KM-40 variant to determine any changes in its genome compared to the parental wild-type strain M-96 of the CMLV. Further, studies on CMI responses are warranted.

Global mass-vaccination campaign with efficacious live attenuated vaccinia virus oral vaccine has led to eradication of smallpox in humans which induced strong immune response both locally and systemic. But orthopoxviruses in domestic animals have been causing outbreaks in many countries. Different types of vaccines such as conventional inactivated/attenuated to recombinant protein-based vaccines have been in use for control of poxvirus infection in animals which are inducing protective and long-lasting immunity. Furthermore, several pox viruses have been in use for generating recombinant vaccines which include vaccinia virus, fowlpox virus, capripoxvirus, parapoxvirus and canary pox for eliciting robust specific cellular and humoral immune responses to the target pathogens (31). Lumpy skin disease (LSD) in cattle and buffalo is caused by a capripox virus and vaccines made of different capripox and sheep pox virus strains have been found effective in inducing immune response and protection against LSD (31).

According to OIE recommendations for live vaccines, it is necessary to conduct studies on the reversion to virulence of attenuated strains by serial passages on susceptible animals (24). We showed that three times passaged attenuated KM-40 strain did not cause any pathology in camels, while the viral DNA was detectable in first and second passages and induced VNA. Interestingly, challenge virulent wild-type virus injected to 1st and 2nd passaged KM-40 strain received animals protected them from illness, whereas the animals in the 3rd passage developed local skin reactions followed by a generalized form of camelpox (data not shown). As recommended by the OIE, the safety of live vaccines is tested in susceptible animals using a large dose of the vaccine (overdose test) (24). Thus, we applied a 20-fold higher selected field dose of KM-40 strain to camels and observed complete safety, with the exception of only a slight increase in body temperature and appearance of small swellings in the skin at the injection site after 6–7 days which disappeared by 10–12 days. Reaction at the injection site is common with other foreign analogs of live vaccines (16–19, 29).

So far until our study there is no chick embryo derived camelpox vaccine. Earlier we demonstrated successful development of a vaccine against camel contagious ecthyma as the most closely related (belonging to the family Poxviridae, genus Parapoxvirus) to camelpox, where the designated virus grew on the CAM of ECE and transferred to the scarified skin of camels (32). In this study, we used similar method of skin scarification for inoculating camels with KM-40 strain and wild-type virus.

It is known that the immunogenicity of vaccines is generally in direct dependence on the dose of the antigen used i.e., higher the antigens in a dose better the immunogenicity and immunity for a long period of time (33, 34). We evaluated efficacy in camels received four doses ranging 10^1.0^-10^4.0^ EID_50_ and found 10^4.0^ EID_50_ appropriate to immunize camels. However, considering the contingencies (climatic conditions, transportation, conditions of use, storage failure, etc.) and high safety profile a 5-fold higher dose of 5 x 10^4.0^ EID_50_ was considered for field use. This dose is several times lower compared to several live vaccines against camelpox [Ducapox vaccine virus strains (10^6.7^ TCID_50_), VD45 (10^5.3^ TCID_50_), CP/NW/92/2 (10^5.6^ TCID_50_) (35), and CMLV/115 (10^5.8^ TCID_50_) (16), except for strain VD47/25 where an immunizing dose is 10^4.7^ TCID_50_ (18). There are no major economic concerns with egg-based vaccines compared to mammalian cell culture-based vaccines. Since more than 70 years egg-based vaccines are being manufactured to make both inactivated influenza (flu shot) and live attenuated vaccines (nasal spray flu vaccine). A minor practical limitation in generating egg-based vaccine for camel pox is the need of obtaining eggs from specific pathogen free birds.

Immunity against camelpox is mediated by both humoral and cellular responses. The relative importance of these two mechanisms is not fully understood, but it is believed that circulating antibodies do not reflect the immune status of the animal (5). In our study, high VNA titers (5.3330 ± 0 ± 0.57 log_2_) were found in camels during early months, but during 10 and 12 months the titers decreased significantly (2.0001 ± 1 ± 0.00 log_2_). Nevertheless, the vaccinated camels were 100% resistant to the experimental infection with wild-type virus even at 12 months after vaccination. This phenomenon can be explained by the fact that immunized animals may have developed cellular immunity in addition to the humoral immune response. It is well documented that in poxvirus infections cellular immunity seems to protect animals from the disease rather than circulating antibodies (36–38).

## Conclusion

We developed a live attenuated egg-based camelpox vaccine from a local field strain in Kazakhstan and evaluated its safety, immunogenicity and protective efficacy in camel. Results of the present study indicated that a novel vaccine derived by serial propagation of KM-40 virus strain on the CAM of the 11-day-old ECE (40 passages) may have the potential to provide protection in camels (*Camelus bactrianus and Camelus dromedaries*) against camelpox.

## Data Availability Statement

The raw data supporting the conclusions of this article will be made available by the authors, without undue reservation.

## Ethics Statement

The animal study was reviewed and approved by The experimental protocol was approved by the Committee on the Ethics of Animal Experiments of the RIBSP of the Science Committee of the Ministry of Education and Science of the Republic of Kazakhstan (permit number: 0414/002 and 0121/013).

## Author Contributions

KT and GJR contributed to the conceptualization, supervision, editing, and project administration. KZh contributed to the writing of the original draft, writing, review, and editing. SK, MM, KZa, MK, YA, YB, MA, and ZA contributed to the animal experiments. KA and RN assisted with the experiments and manuscript preparation. All authors have read and agreed to the current version of the manuscript.

## Conflict of Interest

The authors declare that the research was conducted in the absence of any commercial or financial relationships that could be construed as a potential conflict of interest.

## Publisher's Note

All claims expressed in this article are solely those of the authors and do not necessarily represent those of their affiliated organizations, or those of the publisher, the editors and the reviewers. Any product that may be evaluated in this article, or claim that may be made by its manufacturer, is not guaranteed or endorsed by the publisher.
