# Supplementary material for: Development and Evaluation of a Live Attenuated Egg-Based Camelpox Vaccine
Source: Front Vet Sci. 2021 Aug 16;8:721023. doi: 10.3389/fvets.2021.721023 (PMC8415447; doi:10.3389/fvets.2021.721023)
Supplement: Supplementary file 1 [file Data_Sheet_1.docx]

Supplementary Material

# Preparation of experimental vaccine batch

For the cultivation of attenuated vaccine strain KM-40, 11-day-old embryonated chicken eggs (ECE) were used. Incubated ECE were hand candled before infection. After disinfecting the embryo shell with 70% ethyl alcohol, a hole was pierced over the air pocket with a finely honed metal spear and a window of 4-5 mm in diameter was made with tweezers. A sterile needle was used to inject through the chorioallantoic membrane (CAM) at 2-3 points and the CMLV containing material was applied in a volume of 0.2 mL. The hole in the embryo shell was sealed with a plaster and incubated vertically at (37±0.5) °C for 120 h, depending on the purpose of the experiment, with a relative humidity of (55±5) %. As a control, 4 ECE were injected with the same volume of sterile phosphate buffered saline (PBS). Egg-candling was performed on a daily basis. Embryo death within 48 h was considered nonspecific. Embryos that remained alive for 120 h were cooled in a refrigerator at (4±2) °C for at least 12 h after the incubation period.

Virus-containing materials were collected from the infected ECE after end of the virus cultivation period. To collect virus-containing materials, the shell in the air pocket area was removed with scissors and the CAM was opened. The allantoic fluid was collected into vials, the body and other parts of the embryo were removed from the eggs, CAM was extracted, and if there were any characteristic plaques were present on them, they were placed in a vial containing 30-50 mL (depending on the amount of CAM) of sterile PBS containing antibiotics and kept in the refrigerator for 12 h, homogenized, and frozen at -40 °C. After thawing at room temperature, the CAM suspension was centrifuged at 1000 rpm for 20 min and the homogeneous mass was packed into vials. Allantoic fluid was added to the CAM suspension as needed and frozen at -40 °C, thawed, centrifuged, and the supernatant was used to determine the viral titers. The aliquots of the virus-containing suspension were stored at -40 ºC until further use.

# Determination of the infectious activity of the virus in the ECE

CMLV titer was determined in 11-day-old ECE. Serial 10-fold diluted virus containing material in PBS were prepared. Each dilution of the virus was inoculated into 4 embryos in a volume of 0.1 mL. The infected embryos were labeled with the name of the test material and its dilution rate, and incubated at (37±0.5) °C with (55±5) % relative humidity for up to 7 days. Egg-candling was performed on a daily basis. On the 7th day of incubation, infected ECE were cooled at (4 ± 2) °C for 12 h. The embryos were opened and the presence or absence of characteristic plaques on the CAM were marked with "+" (positive) and no plaques as "-" (negative). The titer of the virus was considered as the highest dilution which caused the characteristic plaques on the CAM in 50% of infected embryos. Virus titer was calculated according to the method of Reed L.J. and Muench H.A.

# Preparation of stabilizing medium and lyophilization

For lyophilization a 13% peptone solution in distilled water was used to stabilize the virus. Peptone was dissolved in a warm water bath; pH of the solution was adjusted to 7.2-7.6 using a 7.5% NaOH solution and sterilized by autoclaving at 121 °C for 15 min. Just before use antibiotics were added to 1 liter of stabilizing medium: benzylpenicillin sodium (potassium) salt – 1,000,000 IU, nystatin – 75,000 IU, and streptomycin sulfate – 500 mg.

# Preparation of 50% glycerol on physiological solution

To immunize animals with lyophilized vaccine against camelpox, a solvent consisting of 50% glycerol solution is required. To prepare 1000 vaccine doses (0.2 mL per dose), 100 mL of glycerol (Sigma-Aldrich, Germany) was dissolved in 100 mL of normal saline, aliquoted into 2 mL (10 doses) glass vials and sealed with sterile rubber plugs with metal caps.

# Lyophilization (freeze drying)

Lyophilization of vaccine virus suspension in ampoules (1 mL) was carried out for 22-24 h in a freeze-drying machine (Labconco, USA) in automatic mode at negative 45-50 °C and vacuum level adjusted at 25-45 Pa in the freeze-drying chamber, and the ampoules were sealed at a residual pressure of 15-30 Pa.
